# MicroRNA-150 modulates intracellular Ca^**2+**^ levels in naïve CD8^+^ T cells by targeting TMEM20

**DOI:** 10.1038/s41598-017-02697-x

**Published:** 2017-06-01

**Authors:** Tae-Don Kim, Hong-Ryul Jung, Sang-Hwan Seo, Se-Chan Oh, Youngho Ban, Xiaoxia Tan, Jung Min Kim, Sang Hyun Lee, Duk-Su Koh, Haiyoung Jung, Young-Jun Park, Suk Ran Yoon, Junsang Doh, Sang-Jun Ha, Inpyo Choi, Philip D. Greenberg

**Affiliations:** 1 Immunotherapy Convergence Research Center, Korea Research Institute of Bioscience and Biotechnology (KRIBB), 125 Gwahak-ro, Yuseong, Daejeon, 34141 Republic of Korea; 2 Department of Functional Genomics, KRIBB School of Bioscience, Korea University of Science and Technology (UST), 217 Gajeong-ro, Yuseong, Daejeon, 34113 Republic of Korea; 3 School of Interdisciplinary Bioscience and Bioengineering (I-Bio), POSTECH, Pohang, 37673 Republic of Korea; 4 Department of Biochemistry, College of Life Science and Biotechnology, Yonsei University, Seoul, Republic of Korea; 5 Departments of Immunology and Medicine, University of Washington School of Medicine and Fred Hutchinson Cancer Research Center, Seattle, Washington, USA; 6 NAR Center, Inc., Daejeon Oriental Hospital of Daejeon University, 22-5 Daeheung-dong, Jung-gu, Daejeon, 34929 Republic of Korea; 7 Department of Physiology and Biophysics, University of Washington School of Medicine, Seattle, Washington, USA

## Abstract

Regulation of intracellular Ca^2+^ signaling is a major determinant of CD8^+^ T cell responsiveness, but the mechanisms underlying this regulation of Ca^2+^ levels, especially in naïve CD8^+^ T cells, are not fully defined. Here, we showed that microRNA-150 (miR-150) controls intracellular Ca^2+^ levels in naïve CD8^+^ T cells required for activation by suppressing TMEM20, a negative regulator of Ca^2+^ extrusion. miR-150 deficiency increased TMEM20 expression, which resulted in increased intracellular Ca^2+^ levels in naïve CD8^+^ T cells. The subsequent increase in Ca^2+^ levels induced expression of anergy-inducing genes, such as *Cbl-b*, *Egr2*, and *p27*, through activation of NFAT1, as well as reduced cell proliferation, cytokine production, and the antitumor activity of CD8^+^ T cells upon antigenic stimulation. The anergy-promoting molecular milieu and function induced by miR-150 deficiency were rescued by reinstatement of miR-150. Additionally, knockdown of TMEM20 in miR-150-deficient naïve CD8^+^ T cells reduced intracellular Ca^2+^ levels. Our findings revealed that miR-150 play essential roles in controlling intracellular Ca^2+^ level and activation in naïve CD8^+^ T cells, which suggest a mechanism to overcome anergy induction by the regulation of intracellular Ca^2+^ levels.

## Introduction

Cluster of differentiation (CD) 8^+^ T cells act as key effector components in adaptive immunity by killing intracellular pathogen-infected and transformed cells. To acquire effector activity, naïve CD8^+^ T cells require triggering via 1) interaction of the T cell receptor (TCR) with antigenic peptide-loaded major histocompatibility complexes, and 2) engagement of co-stimulatory molecules such as CD28. In the absence of co-stimulation, TCR-stimulated naïve CD8^+^ T cells cannot produce interleukin (IL)-2 and proliferate upon subsequent antigenic stimulation. This unresponsiveness of CD8^+^ T cells has been termed anergy^1^.

Activation, as well as anergy induction, of CD8^+^ T cells is associated with Ca^2+^-mediated signaling. TCR stimulation increases intracellular Ca^2+^ concentrations, which induce the nuclear localization of nuclear factor of activated T-cells 1 (NFAT1)^2^. Simultaneously, CD28 co-stimulation activates mitogen-activated protein kinase, which activates activator protein 1 (AP-1). Cooperation of NFAT1 with AP-1 induces expression of genes associated with T cell activation, such as IL-2, whereas NFAT1 activation without AP-1 initiates transcription of anergy-inducing genes, such as early growth-response protein 2 (*Egr2*) and *p27* ^3, 4^. Additionally, naïve T cells can acquire an anergic phenotype if the intracellular Ca^2+^ concentration is sustained at a high level before the TCR stimulation that accompanies co-stimulation, and such hypo-responsiveness can be normalized by blocking Ca^2+^ accumulation. Thus, variations in intracellular Ca^2+^ concentrations may contribute critically to the fate of T cells and the regulation of intracellular Ca^2+^ levels in naïve CD8^+^ T cells should be tightly regulated to avoid T cell anergy^5^. However, the regulating mechanism of intracellular Ca^2+^ in naïve CD8^+^ T cells is not widely defined yet.

During CD8^+^ T cell activation, intracellular Ca^2+^ concentrations are primarily regulated by the calcium release-activated Ca^2+^ (CRAC) channel and plasma membrane Ca^2+^ ATPase (PMCA)^6^. TCR stimulation induces Ca^2+^ release from the endoplasmic reticulum (ER) through the inositol trisphosphate receptor, and the increased cytosolic Ca^2+^ level, in association with calmodulin, activates PMCA to extrude Ca^2+^ from the cell. The reduced Ca^2+^ concentration in the ER lumen, referred to as store depletion, triggers stromal-interacting molecule 1 (STIM1) to open the CRAC channel via interactions with CRAC channel protein 1 (ORAI1) in the plasma membrane. The opened CRAC channel causes an influx of extracellular Ca^2+^ into the cell^7^. Additionally, STIM1 inhibits PMCA-mediated Ca^2+^ removal from the cell^8^. The Ca^2+^-regulating activity of STIM1 is mediated by the formation of a complex with transmembrane protein 20 (TMEM20)^9^, followed by the translocation of the complex to the adjacent side of the plasma membrane, eventually enabling STIM1 to bind PMCA. Based on this Ca^2+^-regulating mechanism, TMEM20 appears to play a crucial role in CD8^+^ T cell activation that must be precisely regulated; however, the specific mechanism by which this occurs and the role in naïve CD8^+^ T cells are largely unknown.

microRNAs (miR) are noncoding RNAs about 22 nucleotides in length that mediate post-transcriptional gene regulation through translational repression and degradation of messenger RNA after binding to the 3′ untranslated region (UTR) of the target RNA^10^. Based on this regulatory activity, miRNAs, including miR-150, are widely associated with the development and biological function of immune cells. During T cell development in the thymus, miR-150 represses neurogenic locus notch-homolog protein 3, a factor involved in T cell differentiation and survival, suggesting that miR-150 is one of the controlling factors for CD8^+^ T cell development^11^. Recently, miR-150 was also shown to be required for CD8^+^ T cell activation^12^, and miR-150-deficient CD8^+^ T cells showed reduced proliferation, differentiation into terminal effector cells, and acquisition of effector function required for killing infected cells. Changes in the transcriptome, such as a decrease in proliferation/killing-associated RNAs and an increase in inhibitory RNAs, were suggested as a molecular mechanism associated with hypo-functional cells; however, the specific mechanism, including the direct target of miR-150 and the molecular events that followed antigenic stimulation in the absence of miR-150 in naïve CD8^+^ T cells, has not been elucidated.

In this study, we revealed that miR-150 is a critical factor in the prevention of an ectopic increase in intracellular Ca^2+^ levels, which prevents anergy induction in naïve CD8^+^ T cells. Mechanistically, miR-150 suppresses increases in intracellular Ca^2+^ levels by directly suppressing TMEM20 expression. This miR-150-induced reduction in TMEM20 allows naïve CD8^+^ T cells to remove intracellular Ca^2+^ and eventually inhibits the expression of anergy-inducing genes, such as Casitas B lineage lymphoma b (*Cbl-b)*, *Egr2*, and *p27*. These findings provide a specific molecular mechanism by which miR-150 regulates CD8^+^ T cell activation and affirms the central importance of intracellular Ca^2+^ regulation in naïve CD8^+^ T cells.

## Results

### miR-150 deficiency increases intracellular Ca^2+^ levels in naïve CD8^+^ T cells

**Figure 1:**
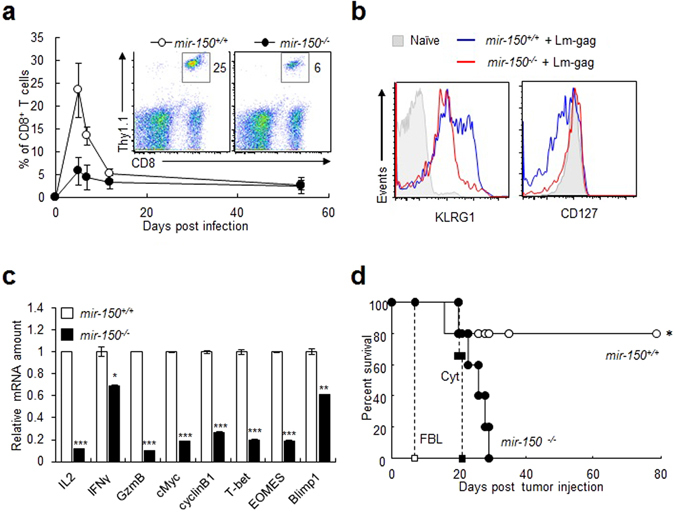
miR-150 deficiency decreases responsiveness to antigenic stimulation and antitumor activity of CD8^+^ T cells *in vivo*. (**a**) Kinetics of *mir-150* ^+/+^ and *mir-150* ^−/−^ CD8^+^ T cells after antigenic stimulation *in vivo*. Thy1.1:TCR_gag_:*mir-150* ^+/+^ or Thy1.1:TCR_gag_:*mir-150* ^−/−^ CD8^+^ T cells were transferred to Thy1.2 C57/BL6 mice. The mice were injected with Lm-gag intraperitoneally. The transferred CD8^+^ T cells were analyzed using blood samples at 0, 5, 7, 12, and 54 d after the transfer. **(b)** Expression of surface markers in *mir-150* ^−/−^ and mir-*150* ^+/+^ CD8^+^ T cells at 5 d after transfer and Lm-gag injection. **(c)** Relative mRNA amount of activation-induced molecules in *mir-150* ^−/−^ and *mir-150* ^+/+^ CD8^+^ T cells after stimulation for 5 h. **(d)** Antitumor activity of *mir-150* ^−/−^ and *mir-150* ^+/+^ CD8^+^ T cells *in vivo*. Mice were injected intra-peritoneally with FBL-gag cell. At 5 d after FBL injection, the mice were received cytoxan (Cyt) and 6 h later transferred *mir-150* ^+/+^ or *mir-150* ^−/−^ CD8^+^ T cells. FBL; FBL injection, Cyt; FBL and Cyt injection, *mir-150* ^−/−^ or *mir-150* ^+/+^; FBL, cyt and CD8^+^ T cell injection. *P < 0.05, **P < 0.01, ***P < 0.001. Data are means ± SEM of duplicate triplicate samples from a single experiment and are representative of two independent experiments.

Since the levels of miR-150 are relatively high in naïve CD8^+^ T cells and gradually decrease after TCR stimulation, miR-150 may be involved in regulating CD8^+^ T cell function^13^. Transferred miR-150-deficient (*mir-150* ^*−/−*^) CD8^+^ T cells showed reduced proliferation *in vivo* (Fig. 1a) after antigen stimulation with recombinant *Listeria*-expressing gag Ag (Lm-gag), and they retained the characteristics of limited differentiation, including low expression of killer cell lectin-like receptor G1 (KLRG1), and high expression of CD127 (Fig. 1b). *mir-150* ^*−/−*^ CD8^+^ T cells also showed reduced function, in terms of decreased expression of effector-associated cytokines [IL-2, interferon-γ (IFN-γ), and granzyme B] and activation-/proliferation-associated molecules (cMyc, cyclin B1, T-bet, EOMES, Blimp1, IL-1β, Klrg1, Cdk6, Cdc23, and Cep120), compared with expression levels observed in *mir-150* ^+/+^ CD8^+^ T cells (Fig. 1c and Fig. S1), which is consistent wit results in a previous report^12^. Notably, the expression of the anergy-inducing gene (*Egr2*) increased in *mir-150* ^*−/−*^ CD8^+^ T cells (Fig. S1c). Additionally, the antitumor activity of CD8^+^ T cells decreased as a result of miR-150 deficiency (Fig. 1d). Collectively, these data suggested that miR-150 is required for proliferation, differentiation, and cytolytic activity of CD8^+^ T cells.

**Figure 2:**
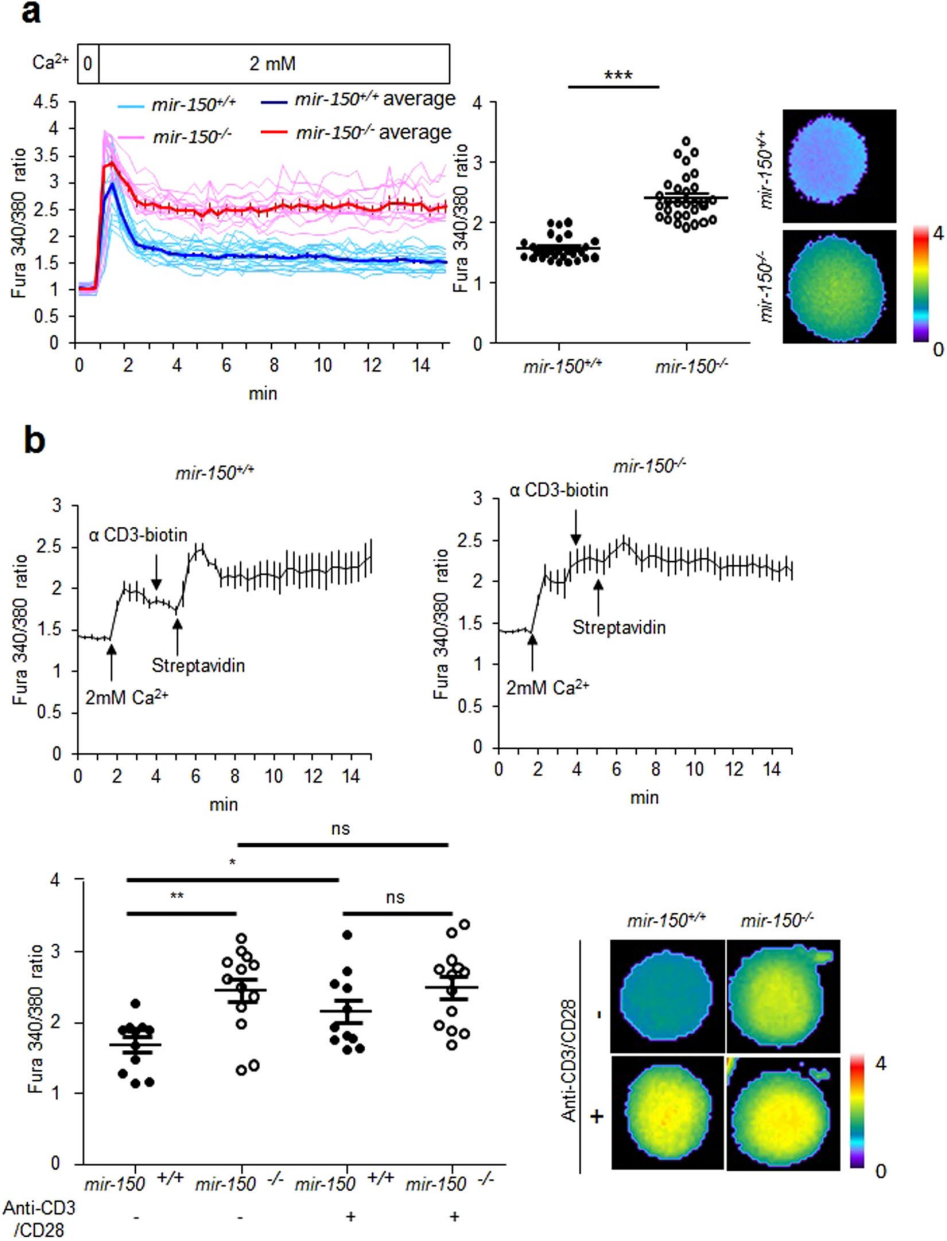
An increased intracellular Ca^2+^ level in naïve CD8^+^ T cells by miR-150 deficiency. (**a**) Comparison of intracellular Ca^**2+**^ levels in *mir-150* ^+/+^ and *mir-150* ^−/−^ naïve CD8^+^ T cells after incubation in media containing 2 mM Ca^**2+**^ (left) and intracellular Ca^2+^ levels at 15 min incubation (middle). For calcium tracing, single cell analyses were performed (sky blue line: *mir-150* ^+/+^ CD8^+^ T cells and pink line: *mir-150* ^−/−^ CD8^+^ T cells) and data were represented with averages (blue line: *mir-150* ^+/+^ CD8^+^ T cells and red line: *mir-150* ^−/−^ CD8^+^ T cells). Images are representative at 15 min after incubation (right). (**b**) The magnitude of increased intracellular Ca^**2+**^ level in *mir-150* ^−/−^ naïve CD8^+^ T cell. *mir-150* ^+/+^ or *mir-150* ^−/−^ naïve CD8^+^ T cells were sequentially treated with anti-CD3-biotin and streptavidin at 4 and 5 min after incubation in Ca^2+^ containing media (up) and intracellular Ca^2+^ levels at 10 min incubation (down left). The images are the representatives at 10 min incubation (down right). *P < 0.05, **P < 0.01, ***P < 0.001, ns: not significant. Data are means ± SEM and are representative of two independent experiments.

To determine the molecular mechanism associated with reduced activation and functionality in *mir-150* ^*−/−*^ CD8^+^ T cells, we first measured intracellular Ca^2+^ levels, because a change in intracellular Ca^2+^ level is one of the initial events during CD8^+^ T cell activation. Naïve *mir-150* ^*−/−*^ CD8^+^ T cells cultured under physiological concentrations of Ca^2+^-containing media exhibited increased intracellular Ca^2+^ levels relative to those in naïve *mir-150* ^+/+^ CD8^+^ T cells (Fig. 2a), and the increased intracellular Ca^2+^ levels in naïve *mir-150* ^−/−^ CD8^+^ T cells were sustained (Fig. S2)^14^. To determine the degree to which intracellular Ca^2+^ levels increased in naïve *mir-150* ^*−/−*^ CD8^+^ T cells, we compared the levels in *mir-150* ^+/+^ and *mir-150* ^*−/−*^ CD8^+^ T cells before and after TCR stimulation. The basal levels of intracellular Ca^2+^ in naïve *mir-150* ^*−/−*^ CD8^+^ T cells were already similar to the increased levels achieved in *mir-150* ^+/+^ CD8^+^ T cells following TCR stimulation and were not further increased by TCR stimulation (Fig. 2b). These data suggested that miR-150 is required for regulation of intracellular Ca^2+^ levels in naïve CD8^+^ T cells.

### miR-150 deficiency leads to expression of anergy-inducing genes through NFAT1 activation in naïve CD8^+^ T cells

**Figure 3:**
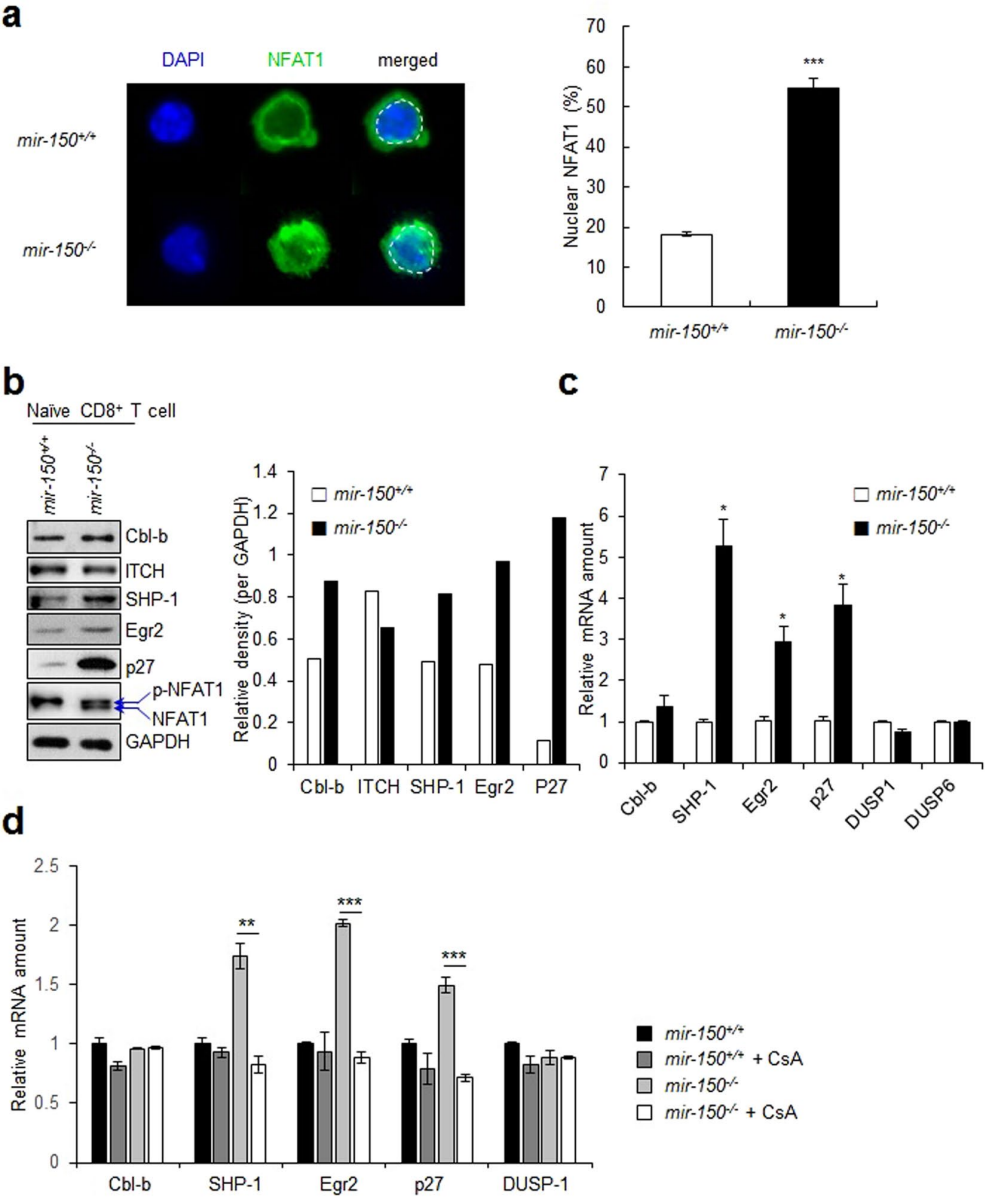
miR-150 deficiency induces the expression of anergy-related genes in naïve CD8^+^ T cells. (**a**) The level of nuclear localization of NFAT1 in *mir-150* ^+/+^ and *mir-150* ^−/−^ naïve CD8^+^ T cells after incubation under Ca^2+^ (2 mM) for at 20 min. The images are the representative samples (left) and the level of nuclear localization of NFAT1 (right). (**b**) The expression level of anergy-related genes and phosphorylation status of NFAT1 in naïve *mir-150* ^+/+^ and *mir-150* ^−/−^ CD8^+^ T cells after incubation in 2 mM Ca^2+^ containing media (left) and the calculated expression levels (right). Western data are the cropped blot images representing indicated proteins. (**c**) Relative expression level of anergy-related and Egr2-related gene in mRNA levels in *mir-150* ^−/−^ naïve CD8^+^ T cells compared with those in *mir-150* ^+/+^ naïve CD8^+^ T cells. (**d**) The effect of inhibition of NFAT translocation on the expression levels of anergy-related genes in *mir-150* ^+/+^ and *mir-150* ^−/−^ naïve CD8^+^ T cells. Isolated *mir-150* ^+/+^ and *mir-150* ^−/−^ naïve CD8^+^ T cells were incubated with or without cyclosporine A (CsA, 3 µM) for 12 h and the amount of anergy-related mRNAs were measured using RT-PCR *P < 0.05, **P < 0.01, ***P < 0.001. Data are means ± SEM of duplicate triplicate samples from a single experiment and are representative of two independent experiments.

Because increased intracellular Ca^2+^ levels in the absence of complete TCR signals are known to activate NFAT1, which can then activate anergy-inducing genes, we hypothesized that the reduced functionality of *mir-150* ^*−/−*^ naïve CD8^+^ T cells might be associated with ectopic activation of NFAT1 in naïve *mir-150* ^*−/−*^ CD8^+^ T cells^2, 5, 15^. Following incubation in physiological Ca^2+^ concentrations, naïve *mir-150* ^*−/−*^ CD8^+^ T cells showed increased nuclear localization of NFAT1 and active forms of NFAT1 (dephosphorylated NFAT1) (Fig. 3a,b). Additionally, naïve *mir-150* ^*−/−*^ CD8^+^ T cells expressed anergy-inducing genes such as *Cbl-b*, Src-homology region 2 domain-containing phosphatase-1 (*SHP-1*), *Egr2*, and *p27*, at higher levels than those observed in naïve *mir-150* ^+/+^ CD8^+^ T cells (Fig. 3b)^4, 16–18^. mRNA levels of anergy-inducing genes, such as *SHP-1*, *Egr2*, and *p27* were also upregulated (Fig. 3c), and treatment with NFAT1 inhibitor (cyclosporine A) reduced the mRNA levels of anergy-related genes (Fig. 3d), indicating that upregulated expression of anergy-inducing genes is associated with NFAT1 activation in naïve *mir-150* ^−/−^ CD8^+^ T cells. Collectively, these results suggested that the reduction in naïve *mir-150* ^−/−^ CD8^+^ T cell function might be associated with the induction of a Ca^2+^-dependent anergy-inducing molecular milieu.

### Elevation of intracellular Ca^2+^ levels in naïve *mir-150*^***−****/****−***^ CD8^+^ T cells is associated with downregulated PMCA activity

**Figure 4:**
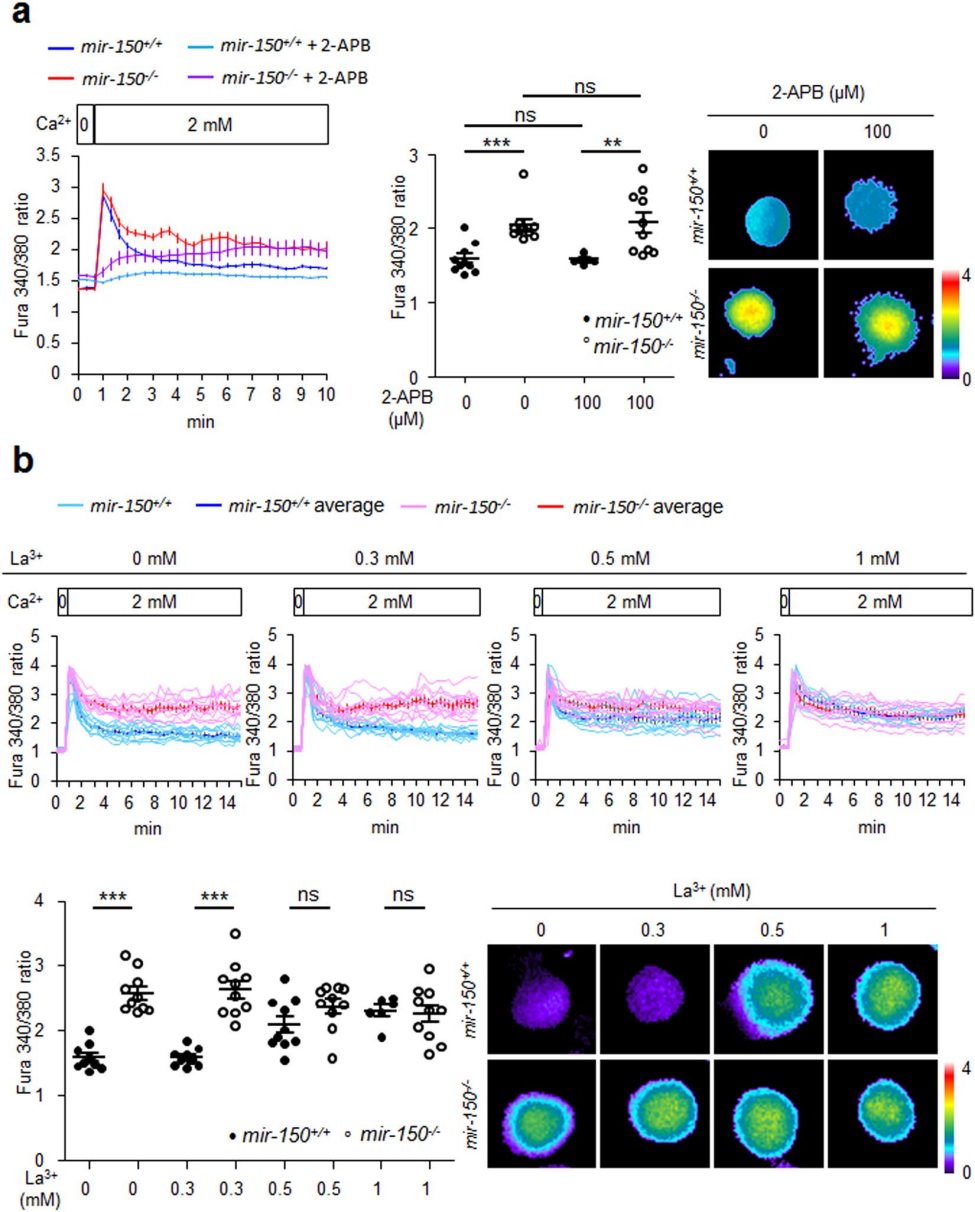
Elevated intracellular Ca^2+^ levels in miR-150 deficient CD8^+^ T cells are caused by down-related PMCA activity. (**a**) Intracellular Ca^2+^ levels in *mir-150* ^+/+^ or *mir-150* ^−/−^ naïve CD8^+^ T cells in the presence or absence of CRAC inhibitor (2-Aminoethoxydiphenyl borate, 2-APB) (left) and the intracellular Ca^2+^ levels at 10 min incubation (middle). Images are the representatives at 10 min incubation (right). *mir-150* ^+/+^ or *mir-150* ^−/−^ naïve CD8^+^ T cells were incubated in 2 mM of Ca^2+^ containing medium in the presence or absence of 2-APB for 10 min and measured the intracellular Ca^2+^ levels from the cells. (**b**) Change in the intracellular Ca^2+^ level by treatment with PMCA inhibitor, La^3+^ (0.3, 0.5 or 1 mM), in *mir-150* ^+/+^ and *mir-150* ^−/−^ naïve CD8^+^ T cells. *mir-150* ^+/+^ and *mir-150* ^−/−^ naïve CD8^+^ T cells were cultures with 2 mM of Ca^2+^ containing medium and incubated in the presence or absence of indicated concentration of La^3+^ (up). Dot graph shows intracellular Ca^2+^ level at 15 min incubation (down left). Images are representatives for the intracellular Ca^2+^ level from the CD8^+^ T cells at 15 min incubation (down right). **P < 0.01, ***P < 0.001, ns: not significant. Data are means ± SEM of duplicate triplicate samples from a single experiment and are representative of two independent experiments.

Intracellular Ca^2+^ concentrations are regulated primarily by CRAC and PMCA^6^. Thus, we first investigated whether the elevated intracellular Ca^2+^ levels in response to miR-150 deficiency were associated with impaired CRAC activity using an inhibitor for CRAC, 2-aminoethoxydiphenyl borate (2-apb). Treatment with 2-apb suppressed the increasing speed in intracellular Ca^2+^ levels both in naïve *mir-150* ^+/+^ and *mir-150* ^−/−^ CD8^+^ T cells (Fig. 4a). However, the intracellular Ca^2+^ levels in naïve *mir-150* ^+/+^ CD8^+^ T cells after 2-apb treatment were similar to those in untreated control CD8^+^ T cells, indicating that CRAC regulates Ca^2+^ uptake in naïve CD8^+^ T cells as it does in TCR-stimulated CD8^+^ T cells, but is not primarily associated with overall intracellular Ca^2+^ levels in naïve CD8^+^ T cells. Therefore, the intracellular Ca^2+^ levels elevated by mir-150 deficiency are not associated with CRAC activity. We then investigated the relationship between the intracellular Ca^2+^ levels elevated by miR-150 deficiency and PMCA function by using two different inhibitors of PMCA activity, La^3+^ and 5(6)-carboxyeosin^19, 20^. La^3+^ treatment elevated intracellular Ca^2+^ levels in a dose-dependent manner in naïve *mir-150* ^+/+^ CD8^+^ T cells, indicating that PMCA activity is crucial to regulating intracellular Ca^2+^ levels in naïve CD8^+^ T cells as it is in TCR-stimulated CD8^+^ T cells (Fig. 4b). The increased Ca^2+^ levels in naïve *mir-150* ^+/+^ CD8^+^ T cells after La^3+^ (1 mM) or 5(6)-carboxyeosin treatment were similar to those in untreated naïve *mir-150* ^−/−^ CD8^+^ T cells (Fig. 4b and Fig. S3). In the case of naïve *mir-150* ^−/−^ CD8^+^ T cells, the intracellular Ca^2+^ levels did not change after treatment with La^3+^ or 5(6)-carboxyeosin. In addition, the calculated Ca^2+^-reducing rates (Fig. 4b) in the untreated set (0 mM La^3+^) were lower in naïve *mir-150* ^−/−^ CD8^+^ T cells than those in naïve *mir-150* ^+/+^ CD8^+^ T cells (Fig. S4), suggesting that PMCA activity might have been impaired in naïve *mir-150* ^−/−^ CD8^+^ T cells before treatment with the PMCA inhibitors.

Mitochondrial calcium uniporter (MCU), sarco/endoplasmic reticulum Ca^2+^-ATPase (SERCA) in the ER, and sodium-calcium exchanger (NCX) in the cellular membrane are also known as regulators of intracellular Ca^2+^ levels^21, 22^. Thus, we also investigated whether these regulators are associated with increased intracellular Ca^2+^ levels in naïve *mir-150* ^−/−^ CD8^+^ T cells. Treatment with inhibitors of MCU (3 mM sodium cyanide + 2ug/ml oligomycin) or SERCA (1uM thapsigargin; TG) did not change intracellular Ca^2+^ levels in naïve *mir-150* ^+/+^ CD8^+^ T cells (Fig. S5). In NCX-inhibited conditions using sodium-free media, intracellular Ca^2+^ levels in naïve *mir-150* ^+/+^ CD8^+^ T cells were similarly unchanged. In naïve *mir-150* ^−/−^ CD8^+^ T cells, the intracellular Ca^2+^ levels did not change in MCU-, SERCA-, or NCX-inhibited conditions. Thus, MCU, SERCA, and NCX are not associated with overall intracellular Ca^2+^ levels in naïve CD8^+^ T cells, suggesting that increased intracellular Ca^2+^ levels in *mir-150* ^−/−^ CD8^+^ T cells are not associated with MCU, SERCA, and NCX. Collectively, these results suggested that increased intracellular Ca^2+^ levels in naïve *mir-150* ^*−/−*^ CD8^+^ T cells may be associated with impaired PMCA activity.

### miR-150 facilitates PMCA function by downregulating TMEM20 expression

**Figure 5:**
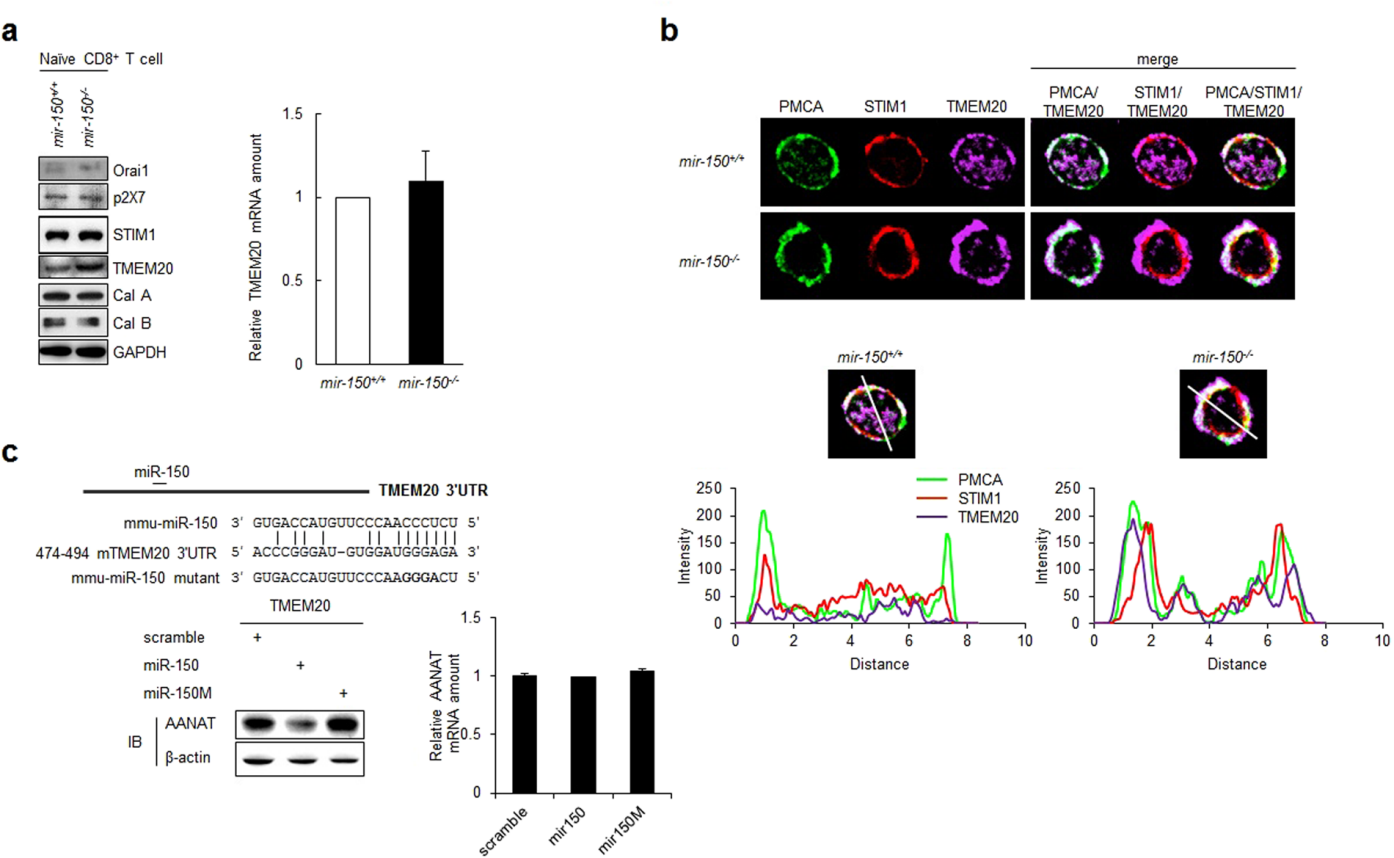
miR-150 specifically binds to 3′UTR of TMEM20 and down-regulates TMEM20 expression. (**a**) The expression level of Ca^2+^-associated molecules in *mir-150* ^+/+^ and *mir-150* ^−/−^ naïve CD8^+^ T cells (left). The relative amount of TMEM20 mRNA in *mir-150* ^+/+^ and *mir-150* ^−/−^ naïve CD8^+^ T cells (right). Western data are the cropped blot images representing indicated proteins. (**b**) Co-localization of STIM1, TMEM20 and PMCA *in mir-150* ^+/+^ and *mir-150* ^−/−^ naïve CD8^+^ T cells (up) and the fluorescence intensity for each molecules on the line (down). Green; PMCA, Red; STIM1, Purple; TMEM20. (**c**) Post-transcriptional regulation of miR-150 by targeting 3′UTR of TMEM20. miR-150 binding sequences of TMEM20 3′UTR (left up). The level of AANAT protein (left down) and mRNA (right) in NIH3T3 cells co-transfected with plasmid-expressing AANAT reporter containing TMEM20 3′UTR and miR-150 or miR-150 mutant. Western data are the cropped blot images representing indicated proteins. *P < 0.05. Data are means ± SEM of duplicate triplicate samples from a single experiment and are representative of two independent experiments.

Because PMCA activity is negatively regulated by the STIM1-TMEM20 complex^9^, we investigated whether the impaired PMCA activity in *mir-150* ^*−/−*^ naïve CD8^+^ T cells was associated with altered expression of STIM1 and/or TMEM20. TMEM20 protein expression was upregulated in *mir-150* ^*−/−*^ naïve CD8^+^ T cells, but STIM1 and other Ca^2+^-regulation-associated molecules were unchanged (Fig. 5a). Confocal microscopic analysis also showed that TMEM20 expression was higher and co-localization of TMEM20 with PMCA was greater in naïve *mir-150* ^*−/−*^ CD8^+^ T cells than those in naïve *mir-150* ^+/+^ CD8^+^ T cells (Fig. 5b and Fig S6). Interestingly, TMEM20 did not seem to co-localize with STIM1 in both naïve *mir-150* ^+/+^ and *mir-150* ^*−/−*^ CD8^+^ T cells, indicating that the increased intracellular Ca^2+^ levels in *mir-150* ^−/−^ CD8^+^ T cells were not associated with STIM1. Thus, TMEM20 may suppress PMCA activity in a STIM1-independent manner in naïve *mir-150* ^−/−^ CD8^+^ T cells.

To assess the potential for miR-150 to suppress TMEM20 expression specifically, we performed an arylalkylamine *N*-acetyltransferase (AANAT)-reporter assay as previously described^23, 24^. Based on the miRanda algorithm (www.microrna.com), the 3′UTR of TMEM20 mRNA contains a putative binding sequence for miR-150 (Fig. 5c), and overexpression of miR-150 reduced the expression of the AANAT reporter protein but not AANAT mRNA. In contrast, forced expression of a miR-150 mutant incapable of binding to the TMEM20 3′UTR did not change AANAT expression, suggesting that miR-150 suppresses the expression of TMEM20, and has the potential to do so directly.

### miR-150 restoration recovers the miR-150 phenotype in miR-150-deficient CD8^+^ T cells

**Figure 6:**
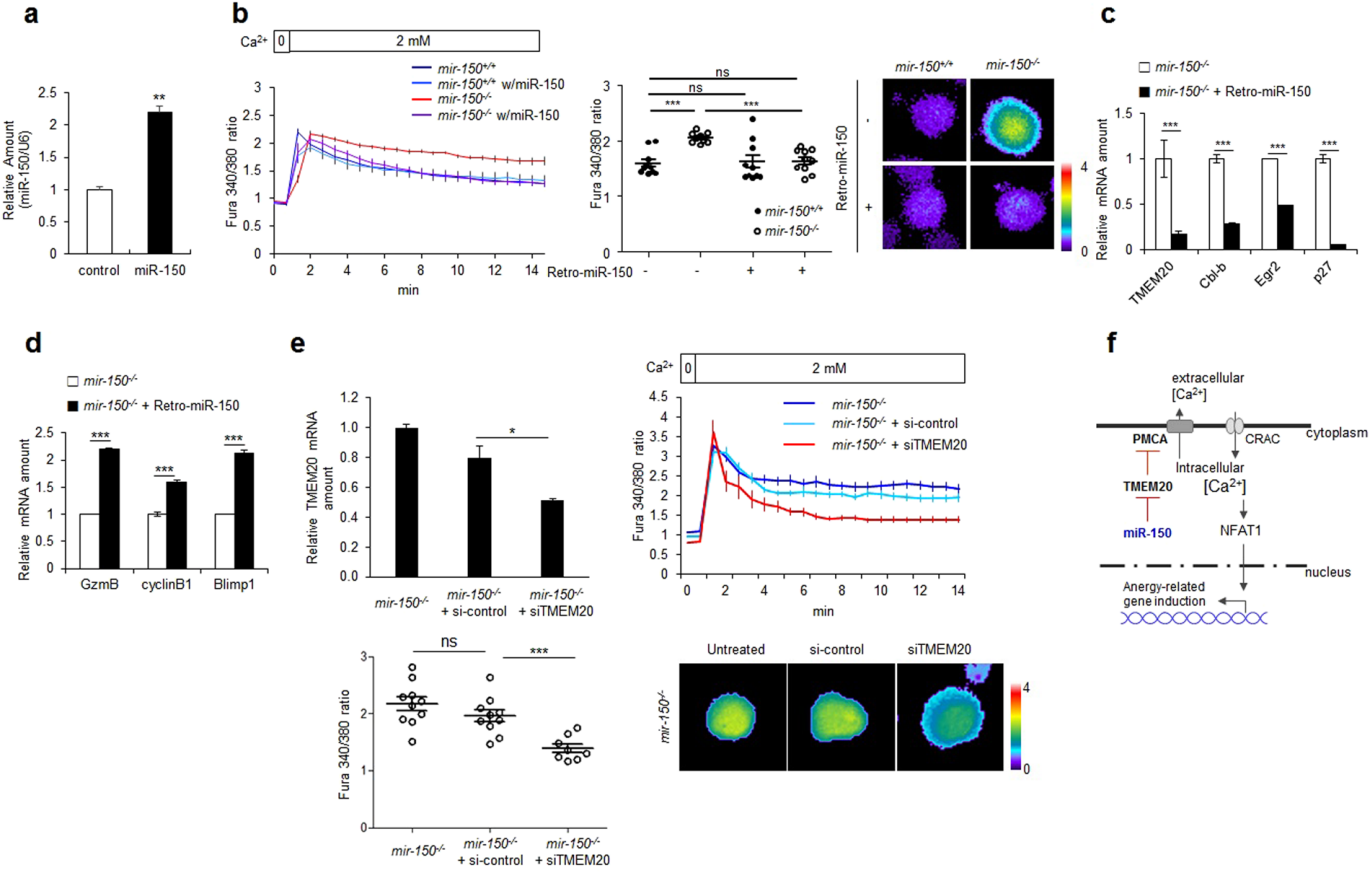
Add-back of miR-150 rescues Ca^2+^ homeostasis and inhibits expression of anergy-related genes in *mir-150* ^−/−^ naïve CD8^+^ T cells. (**a**) Isolated *mir-150* ^+/+^ naïve CD8^+^ T cells were infected with 10 MOI of retrovirus co-expressing miR-150 and GFP (retro-miR-150-gfp) and the miR-150 levels were analyzed. (**b**) Intracellular Ca^2+^ levels in retro-miR-150 transduced or control *mir-150* ^+/+^ or *mir-150* ^−/−^ naïve CD8^+^ T cells (left). Dot graph shows intracellular Ca^2+^ levels at 15 min incubation (middle) and the representative images (right). (**c**) Expression levels of TMEM20 and anergy-related genes after add-back of miR-150 by transduction of retro-miR-150 in *mir-150* ^−/−^ naïve CD8^+^ T cells. (**d**) Relative expression level of granzyme B, cyclinB1 and Blimp1 after stimulation using anti-CD3/CD28 in *mir-150* ^−/−^ CD8^+^ T cells and retro-miR-150 infected *mir-150* ^−/−^ CD8^+^ T cells. (**e**) TMEM20 mRNA amount (up left) and intracellular Ca^2+^ levels (up right) in siRNA for TMEM20 or control-siRNA transfected *mir-150* ^−/−^ naïve CD8^+^ T cells (left). Dot graph shows intracellular Ca^2+^ levels at 10 min incubation (down left) and representative images (down right) (**f**) Schematic cartoon for the effect of miR-150 on Ca^2+^ homeostasis and induction of anergy-related genes in naïve CD8^+^ T cells. *P < 0.05, **P < 0.01, ***P < 0.001, ns: not significant.

We then investigated whether miR-150 restoration could recover intracellular Ca^2+^ levels and the molecular signature in naïve *mir-150* ^*−/−*^ CD8^+^ T cells. We infected naïve *mir-150* ^*−/−*^ CD8^+^ T cells with a retrovirus-expressing miR-150 (retro-miR-150) and analyzed the resulting cell phenotype. miR-150 restoration increased miR-150 levels in CD8^+^ T cells (Fig. 6a). Furthermore, retro-miR-150 infection reduced intracellular Ca^2+^ levels in *mir-150* ^*−/−*^ CD8^+^ T cells to levels observed in *mir-150* ^+/+^ CD8^+^ T cells (Fig. 6b). However, intracellular Ca^2+^ levels in *mir-150* ^+/+^ CD8^+^ T cells were unchanged by the additional expression of miR-150, whereas TMEM20 expression in *mir-150* ^*−/−*^ CD8^+^ T cells was reduced by restoration of miR-150 (Fig. 6c). Additionally, retro-miR-150 infection of *mir-150* ^*−/−*^ CD8^+^ T cells reduced expression of anergy-related genes *Cbl-b*, *Egr2*, and *p27* and increased the expression of activation-induced molecules granzyme B, cyclin B1, and Blimp1 following anti-CD3/CD28 stimulation (Fig. 6c,d). In addition, suppression of TMEM20 expression by infection with small interfering RNA targeting TMEM20 (siTMEM20) decreased intracellular Ca^2+^ levels in naïve *mir-150* ^*−/−*^ CD8^+^ T cells, indicating that the balance between TMEM20 and miR-150 regulates intracellular Ca^2+^ levels in naïve CD8^+^ T cells (Fig. 6e). These data suggested that miR-150 is required for activation of naïve CD8^+^ T cells by regulating intracellular Ca^2+^ levels and preventing the expression of anergy-related genes (Fig. 6f).

## Discussion

In CD8^+^ T cells, increasing the concentration of intracellular Ca^2+^ after TCR stimulation is essential to inducing the expression of activation-associated genes^2, 25, 26^. Because controlling the Ca^2+^ concentration is crucial for T cell activation, there has been an extensive search for molecules, such as channels, transporters, and other proteins, associated with Ca^2+^ movement during T cell activation. The preconditions for increasing intracellular Ca^2+^ levels during T cell activation are a relatively low level of intracellular Ca^2+^ in naïve CD8^+^ T cells and an ectopic increase in intracellular Ca^2+^ levels before T cell activation turns T cells into tolerant cells^5^. Although controlling intracellular Ca^2+^ levels in naïve CD8^+^ T cells is also critical for T cell activation, regulatory molecules and associated mechanisms that determine the Ca^2+^ level in naïve CD8^+^ T cells are largely unknown. In this study, we showed that miR-150 controls intracellular Ca^2+^ levels by downregulating TMEM20 expression in naïve CD8^+^ T cells. The miR-150-induced suppression of TMEM20 expression prevented the expression of anergy-inducing genes and hypo-responsiveness of CD8^+^ T cells upon antigenic stimulation. Therefore, miR-150 is an essential regulator that creates an activation-favorable molecular milieu in naïve CD8^+^ T cells.

In this study, we showed that intracellular Ca^2+^ levels are increased by miR-150 deficiency in naïve CD8^+^ T cells. In *mir-150* ^−/−^ naïve CD8^+^ T cells, the expression level of TMEM20 was increased, while expression levels of other Ca^2+^ regulating molecules did not changed. In addition, suppression of TMEM20 decreased intracellular Ca^2+^ levels in *mir-150* ^−/−^ naïve CD8^+^ T cells, indicating that the increased intracellular Ca^2+^ levels in *mir-150* ^−/−^ naïve CD8^+^ T cells are derived from the increased expression of TMEM20. Previous report showed that TMEM20 attenuates PMCA activity^9^. *mir-150* ^−/−^ naïve CD8^+^ T cells showed high translocation of TMEM20 into PMCA in plasma membrane. In addition, Ca^2+^ reducing rate also decreased in *mir-150* ^−/−^ naïve CD8^+^ T cells. Treatment of PMCA inhibitor did not changed the intracellular Ca^2+^ level in *mir-150* ^−/−^ naïve CD8^+^ T cells whereas treatment of inhibitors for other Ca^2+^-regulating molecules changed the intracellular Ca^2+^ levels in *mir-150* ^−/−^ naïve CD8^+^ T cells, which also can be an indirect clue that PMCA activity is inactivated in *mir-150* ^−/−^ naïve CD8^+^ T cells. Collectively, the high Ca^2+^ levels in *mir-150* ^−/−^ naïve CD8^+^ T cells are derived from increased expression of TMEM20, thereby inactivation of Ca^2+^ extruding PMCA activity.

Several regulators such as PMCA, CRAC, SERCA, NCX, and MCU control intracellular Ca^2+^ levels in TCR-stimulated CD8^+^ T cells^22, 27^. In this study, we showed that PMCA, but not other regulators, mainly controls intracellular Ca^2+^ levels in naïve CD8^+^ T cells. Although CRAC inhibition slowed the Ca^2+^ uptake rate in naïve CD8^+^ T cells, overall intracellular Ca^2+^ levels were not changed, indicating that the role of CRAC is limited to regulating intracellular Ca^2+^ level in naïve CD8^+^ T cells. The unchanged intracellular Ca^2+^ levels in naïve CD8^+^ T cells in SERCA- and NCX-inactivated conditions could be a result of Ca^2+^-regulating activity that is TCR-activation dependent. For MCU, their Ca^2+^-regulating function is associated with relocation of mitochondria. In TCR-stimulated T cells, mitochondria relocate to the immunological synapse, where the mitochondria act as Ca^2+^-controlling machinery through MCU^21^. Un-polarized mitochondria at the sites of Ca^2+^ influx in naïve CD8^+^ T cells might be a reason that MCU is not associated with intracellular Ca^2+^ levels in naïve CD8^+^ T cells.

In a previous study, TMEM20-mediated suppression of PMCA activity was shown to be dependent on calcium store depletion in the ER and co-localization with STIM1 in a Jurkat cell line model^9^. However, our co-localization data showed that TMEM20 does not co-localize with STIM1 either in naïve *mir-150* ^+/+^ or *mir-150* ^−/−^ CD8^+^ T cells. In addition, the expression levels of STIM1 were similar between naïve *mir-150* ^+/+^ and *mir-150* ^−/−^ CD8^+^ T cells. Considering the increased intracellular Ca^2+^ levels in naive *mir-150* ^−/−^ CD8^+^ T cells, STIM1 does not act as a regulator of intracellular Ca^2+^ in naïve CD8^+^ T cells. Thus, PMCA may act in a STIM-independent manner in naïve CD8^+^ T cells. Given that mir-150 has many targets, expression of undefined factor(s) supporting TMEM20 function could be increased in naïve *mir-150* ^−/−^ CD8^+^ T cells, and, thereby, the increase in TMEM20 with the associated factor(s) might suppress PMCA activity in naïve *mir-150* ^−/−^ CD8^+^ T cells before calcium store-depletion in the ER. Another possible mechanism for calcium store depletion independent of TMEM20-mediated PMCA deactivation is TMEM20 expression in the plasma membrane, which might deactivate PMCA function itself, but its function could also be limited by insufficient expression to act in naïve *mir-150* ^+/+^ CD8^+^ T cells. In this case, increased TMEM20 expression in naïve *mir-150* ^−/−^ CD8^+^ T cells may be sufficient to deactivate PMCA function, resulting in the increase in intracellular Ca^2+^ levels in naïve *mir-150* ^+/+^ CD8^+^ T cells.

The expression levels of TMEM20 mRNA were similar between naïve *mir-150* ^+/+^ and *mir-150* ^−/−^ CD8^+^ T cells. However, retroviral-mediated overexpression of miR-150 downregulated the expression of TMEM20 mRNA. Considering that miR-150 levels in retro-miR-150-infected *mir-150* ^+/+^ CD8^+^ T cells were more than 2-fold higher than those in the uninfected control *mir-150* ^+/+^ CD8^+^ T cells, retro-miR-150 infected *mir-150* ^−/−^ CD8^+^ T cells may have higher levels of miR-150 than those in *mir-150* ^+/+^ CD8^+^ T cells. Thus, it is possible that a normal amount of miR-150, such as that in naïve *mir-150* ^+/+^ CD8^+^ T cells, might repress translation of TMEM20, whereas overexpression of miR-150, such as that in retro-miR-150 infected naïve *mir-150* ^−/−^ CD8^+^ T cells, might suppress transcription of TMEM20. Given that miR-150 has many targets, miR-150 overexpression might induce suppression of TMEM20 as well as have additional or alternative effects, and the additional effects, not suppression of TMEM20 levels, could be the reason for the decreased intracellular Ca^2+^ levels in retro-miR-150-infected naïve *mir-150* ^−/−^ CD8^+^ T cells. However, we showed that suppression of TMEM20 expression decreased the intracellular Ca^2+^ levels in naïve *mir-150* ^−/−^ CD8^+^ T cells. Thus, TMEM20 expression levels, especially in the absence of miR-150, are critical to controlling intracellular Ca^2+^ levels in naïve CD8^+^ T cells.

Whether simply overexpressing TMEM20 is sufficient to increase intracellular Ca^2+^ levels in naïve *mir-150* ^+/+^ CD8^+^ T cells in questionable. In a previous report, TMEM20 overexpression did not change the intracellular Ca^2+^ levels in HEK 293 cells expressing STIM1 and Orai1 in thapsigargin-treated conditions, but thapsigargin-untreated conditions were not tested^9^. Although we showed that miR-150 deficiency did not change the expression levels of Ca^2+^-regulating molecules, except for TMEM20, miR-150 deficiency may influence undefined factor(s) required for TMEM20 function, as described above. In this case, simply overexpressing TMEM20 might not increase intracellular Ca^2+^ level in naïve *mir-150* ^+/+^ CD8^+^ T cell. To date, the mechanism of TMEM20 function and its associated/regulating molecules have not been widely investigated. Thus, studies on the mechanism of TMEM20 function and regulation should be conducted to better understand the detailed mechanism of Ca^2+^ regulation in naïve CD8^+^ T cells before and after activation.

miR-150 deficiency reduces CD8^+^ T cell activation, in terms of expansion, differentiation, and cytolytic function^12^. Although changes in the levels of several mRNAs in response to miR-150 deficiency were suggested in a previous study to be a mechanism for the observed reduction in CD8^+^ T cell activation, a direct relationship between altered mRNA levels and CD8^+^ T cell activation-associated molecular changes was not found. In this study, we showed that TMEM20 is a direct target for miR-150 in naïve CD8^+^ T cells and that the miR-150 deficiency-induced reduction in CD8^+^ T cell activation results from altered intracellular Ca^2+^ levels. In the absence of miR-150, elevated expression of TMEM20, a Ca^2+^ extruder, attenuates PMCA activity, resulting in increased intracellular Ca^2+^ levels in naïve CD8^+^ T cells^8, 9^. This increased intracellular Ca^2+^ induced activation of NFAT1, which consequently induced expression of anergy-inducing genes such as *Egr2*, *Cbl-b*, and *p27* ^16, 17, 28^. Because anergy-inducing genes such as *p27* and *Egr2* are reportedly miR-150 targets, increased expression of *p27* and *Egr2* in *mir-150* ^−/−^ CD8^+^ T cells may be a result of the loss of translational suppression of these mRNAs by miR-150 in *mir-150* ^*−/−*^ naïve CD8^+^ T cells^29, 30^. However, following treatment with an NFAT1 inhibitor, *mir-150* ^*−/−*^ naïve CD8^+^ T cells showed decreased levels of *p27* and *Egr2* mRNA, indicating that increased expression of anergy-inducing genes is primarily due to transcriptional regulation via the intracellular Ca^2+^/NFAT1 signaling pathway. Expression of these anergy-inducing genes related to miR-150 deficiency could explain the reduced proliferation, differentiation, and killing activity of naïve *mir-150* ^−/−^ CD8^+^ T cells upon antigenic stimulation, as previously reported^12^ and shown in our study. To avoid these miR-150-deficiency-induced molecular events, intracellular Ca^2+^ levels need to be sufficiently low to prevent NFAT1 activation.

It was previously reported that in effector CD8^+^ T cells, miR-150 levels are lower than in naïve CD8^+^ T cells^13^. Given that miR-150 suppresses expression of CD25, an IL-2 receptor, reduced miR-150 levels in effector CD8^+^ T cells can favor survival and proliferation^31^. Relatively low levels of miR-150 in effector CD8^+^ T cells may also be explained by intracellular Ca^2+^-associated events. Because miR-150 deficiency elevated intracellular Ca^2+^ levels, decreased levels of miR-150 in effector CD8^+^ T cells implies that effector CD8^+^ T cells may require higher intracellular Ca^2+^ concentrations to promote activity such as that of cytotoxic T lymphocytes (CTLs). Previous reports showed that intracellular Ca^2+^ is involved in granule reorientation toward the target cell contact region and exocytosis of granules^32–34^. Additionally, intracellular Ca^2+^ mediates the production of effector cytokines and the expression of death receptor ligands^35–37^. To mediate these diverse functions associated with CTLs, high intracellular Ca^2+^ concentrations may be required, which might be satisfied by miR-150 downregulation in effector CD8^+^ T cells.

miR-150 acts as a “fine tuner” of various immune cells. In B cells, miR-150 controls differentiation and receptor signaling^38–40^. In natural killer cells, miR-150 regulates development, as well as cytotoxicity^24, 41^. T cell differentiation and the expression of cytokine receptors are also influenced by miR-150^11, 31^. Here, we showed that miR-150 controlled Ca^2+^ signaling and prevented the induction of anergy in naïve CD8^+^ T cells. Considering these various roles for miR-150 in immune cells and various target molecules, studies to determine additional miR-150 roles in the immune system will likely shed light on currently undefined mechanisms as well as possible therapeutic approaches using CD8^+^ T cells.

Given the impact of miR-150 on CD8^+^ T cell activation, regulation of miR-150 expression may be a therapeutic target in CD8^+^ T cell-associated diseases. Because we showed that *mir-150* ^*−/−*^ naïve CD8^+^ T cells could not be activated, CD8^+^ T cell-specific suppression of miR-150 expression may be a novel approach to treating autoimmune diseases. In this context, our findings indicate a molecular mechanism that prevents the transition of CD8^+^ T cells into a hypo-responsive state, as well as a basis for regulation of CD8^+^ T cell activation.

## Methods

### Mice

TCR_gag_ transgenic mice have been generated as previously described^42^. C57BL/6 (B6) mice and *mir-150* ^−/−^ mice on a B6 background were purchased from The Jackson Laboratory and TCR_gag_:*mir-150* ^+/+^ and TCR_gag_:*mir-150* ^*−/−*^ mice were generated in our animal facility. All mice were bred and maintained under Specific Pathogen Free conditions. All of the methods and experimental procedures were conducted according to the approved (approval ID: KRIBB-AEC-15088) guidelines and regulations by the animal ethics committee (IACUC) of KRIBB, South Korea.

### Cell lines

The Friend virus-induced erythroleukemia of B6 origin, FBL, expresses the FMuLV-encoded gag epitope (peptide CCLCLTVFL purchased from Pi Proteomics), and was maintained in culture in RPMI1460 supplemented with 10% FBS and antibiotics. NIH3T3 cells for plasmid transfection were cultured in DMEM supplemented with 10% FBS and antibiotics.

### Expansion of effector TCR_gag_ CD8^+^ T cells *in vitro*

TCR_gag_:*mir-150* ^+/+^ and TCR_gag_:*mir-150* ^*−/−*^ CD8^+^ T cells (1 × 10^6^) were cultured with irradiated syngeneic splenocytes (5 × 10^6^), irradiated FBL-gag leukemia (3 × 10^6^), and IL-2 (20 U/ml) in RPMI 1640 supplemented with 2 μM glutamine, 100 U/ml penicillin/streptomycin, 10% FCS, and 30 μM 2-mercaptoethanol. Effector CD8^+^ T cells after five days following antigen stimulation were used in various assays or transferred for adoptive immunotherapy.

### T cell proliferation *in vivo*

Thy1.1^+^ CD8^+^ donor cells (1 × 10^6^) from TCR_gag_:*mir-150* ^+/+^ and TCR_gag_:*mir-150* ^*−/−*^ mice were transferred into Thy1.2^+^ B6 recipients. At one day after the transfer, the mice were injected with recombinant attenuated (Δ*actA*, Δ*inlB*) *Listeria monocytogenes* expressing the FMuLV GAG epitope (CCLCLTVFL) (Lm-gag) or a control vector (Lm-Ø) (kindly provided by Aduro BioTech, Inc) intraperitoneally. T cell persistence and proliferation were monitored in peripheral blood.

### Immunofluorescence

Glass coverslips-attached cells were stimulated with CaCl_2_, PMA/Ionomycin, and anti-CD3 for indicated time points, washed using PBS, fixed in 3.7% formaldehyde for 10 min at 37 °C, permeabilized with 0.2% Triton X-100 for 10 min at room temperature, washed, and then blocked in 1% BSA in PBS for 30 min. To visualize NFAT1, the cells were stained with anti-NFAT1 (Abcam) for 1 h, washed and incubated with FITC-conjugated anti-mouse antibody (Santa Cruz Biotechnology) for 1 h. Images were captured with an Olympus DP30BW digital camera and processed using the *Metamorph* 7.1 program (Universal Imaging, Media, PA, USA). Nuclear translocation of NFAT1 was measured by percentage of NFAT1 localized in the nucleus.

### Adoptive immunotherapy of disseminated FBL leukemia

B6 mice were injected with FBL-gag leukemia cells (5 × 10^6^) intraperitoneally (i.p.) as described^16^. At 5 days later, mice received cytoxan (Cyt; 180 mg/kg, R&D systems) i.p., and 6 h later, received TCR_gag_:*mir-150* ^+/+^ or *mir-150* ^*−/−*^ effector CD8^+^ T cells intravenously.

### Measurement of intracellular Ca^2+^ in CD8^+^ T cells

CD8^+^ T cells were loaded with 2 μM fura-2/AM (Life Technologies) for 20 min at 37 °C and attached to poly-L-lysine treated glass cover slips loaded in a Chamlide magnetic chamber (Live Cell Instrument) under Ca^2+^-free HSBB medium. The fura-2 loaded cells were perfused using HBSS medium containing 2 mM Ca^2+^. Cell images were obtained on a Zeiss Axio Observer.Z1 epifluorescence microscope. Fura-2 emission was detected at 510 nm after excitation at 340 and 380 nm. In some experiments, biotin-conjugated anti-CD3 (5 μg/ml, 2C11, BioXCell), anti-CD28 (5 μg/ml, 37.51, BioXCell) and streptavidin (10 μg/ml, Thermo Scientific) were added at the indicated time. To measure intracellular Ca^2+^ concentration in the CRAC, PMCA, SERCA, or MCU inhibited conditions, CD8^+^ T cells were pre-treated with specific inhibitors as below and measured intracellular Ca^2+^ levels as described above. CRAC inhibitor: 2-aminoethoxydiphenyl borate (2-APB, 100 uM, Sigma-Aldrich), PMCA inhibitors: La^3+^ (0.3, 0.5 or 1 mM, Sigma-Aldrich) and 5(6)-carboxyeosin (20 uM, AXXORA), SERCA inhibitor: thapsigargin (1 µM, Sigma-Aldrich), MCU inhibitor: sodium cyanide (3 mM, Sigma-Aldrich) + oligomycin (2 µg/mL, Sigma-Aldrich). For NCX inhibition, Na-free media (ThermoScientific) was used. Acquired time-lapse images and Fura 340/380 ratio images were analyzed with ImageJ software (National Institutes of Health) and Metamorph (Molecular Devices).

### Flow cytometry

All antibodies for flow cytometric analysis were purchased from Becton Dickinson or BD Pharmingen. CD8^+^ T cells were stained with the indicated antibodies in a staining buffer (PBS containing 1% FBS and 0.01% NaN3) for 20 minutes at 4 °C. After washing, flow cytometry was performed on a BD FACS Canto II and data analyzed with FlowJo software (Tree Star).

### Immunoblot analysis

Cytoplasmic lysates of CD8^+^ T cells were extracted with PBS containing 1% NP-40 and complete proteinase inhibitor (Roche). Immunoblot analyses were performed with primary antibodies purchased from Abcam (anti-ITCH and anti-Egr2), Cell Signaling (anti-Calcineurin A, anti-NFAT1, anti-p27, and anti-STIM1), Santa Cruz Biotech (anti-Calcineurin B, anti-Cbl-b, anti-SHP-1, and anti-TMEM20), GenTex (anti-Orai1), Thermo Scientific (anti-P2X7) and AbFRONTIER (anti-GAPDH) and gifted from Dr. Klein DC (anti-AANAT). The secondary antibodies were visualized using an Immobilon Western kit (MILLIPORE).

### Reverse transcription and quantitative PCR

Total RNA extracted with TRIzol (Invitrogen) was reverse transcribed with Moloney murine leukemia virus reverse transcriptase and oligo-d(T). For real-time qPCR, cDNA was amplified with specific primers and SYBR Premix Ex Taq (Takara Bio) on a Dice TP800 Thermal Cycler (Takara Bio). The mRNA levels are presented relative to expression of GAPDH mRNA. Quantitative real-time PCR of mature miR was performed with a TaqMan MicroRNA Assay kit (Applied Biosystems) as previously described^23, 24^. miR expressions are presented relative to the level of U6 small nuclear RNA.

### mRNA array and data analysis

To collect naïve, effector, and memory CD8^+^ T cells, mice infected with Lm-gag as described in “T cell proliferation *in vivo*”, samples were isolated from the spleen at day 0, 5, and 50 after Lm-gag infection, respectively, and analyzed the phenotype of each samples (naïve: CD8^+^CD44^lo^CD62L^hi^, effector: CD8^+^CD44^hi^CD62L^lo^, and memory: CD8^+^CD44^hi^CD62L^hi^). Labeling of target mRNAs and hybridization were performed using Agilent’s mRNA Labeling Reagent and Hybridization Kit (Agilent Technology) as manufacturer’s instruction. The hybridization images were analyzed with an Agilent DNA microarray Scanner and quantified by using Agilent Feature Extraction software. Normalization and selection of all data were performed by using GeneSpring GX 7.3 (Agilent Technology). For microarray data, genes were filtered with removing flag-out genes in each experiment. In the gene expression microarray, intensity-dependent normalization (LOWESS) was performed, where the ratio was reduced to the residual of the Lowess fit of the intensity vs. ratio curve. The averages of normalized ratios were calculated by dividing the average of normalized signal channel intensity by the average of normalized control channel intensity. Functional annotation of genes was performed according to Gene Ontology^TM^ Consortium (http://www.geneontology.org/index.shtml) by GeneSpring GX 7.3. Gene classification was based on searches done by GeneCards (http://www.genecards.org/), miRanda (http://www.microrna.org/), DAVID (http://david.abcc.ncifcrf.gov/), and Medline databases (http://www.ncbi.nlm.nih.gov/). The microarray data can be accessed from the Gene Expression Omnibus under the accession **GSE62262**.

### Plasmids and Reporter assay

To construct chimeric reporter plasmids pcAANAT-TMEM20, 3′UTR of the mouse TMEM20 was amplified by DNA polymerase (TAKARA) using specific primers (forward: 5′-AAGAATTCTGAAGTGTCACTGCTGA-3′ and reverse: 5′-AACTCGAGAGTTGTAAAACCCA-ACA-3′) and confirmed by sequencing. For AANAT reporter assays, NIH3T3 cells were co-transfected with 1 µg of pcAANAT- TMEM20 plasmids with one of either the miR-150 mimics, miR-150 mutants, or the non-targeting control (BiONEER) at concentrations of 50~100 nM using TransIT-X2 (Mirus Bio LLC). Transfected cells were further grown for 48 h before harvesting.

### Co-localization measurement of STIM1, PMEM20, and PMCA

Isolated CD8^+^ T cells were incubated in the presence of 2 mM of Ca^2+^, fixed with paraformaldehyde (Sigma-Aldrich), permeabilized using Triton X-100 (Sigma-Aldrich) and stained with anti-PMCA (Abcam), anti-TMEM20 (SantaCruz Biotechnology) or anti-STIM1 (Cell Signaling Technology) antibody. Then the samples were stained with anti-rabbit IgG-Alex Fluor^®^ 488, anti-rabbit-IgG-Alex Fluor^®^ 546 or IgG-Alex Fluor^®^ 647 (Life Technologies, Gaithersburg, MD) for PMCA, STIM1 or TMEM20, respectively. The images were obtained using LSM 510 META Laser Scanning Microscopes (Carl Zeiss).

### Retrovirus preparation and transduction

pMXs-miR-GFP/puro retroviral expression vector was purchased from Cell Biolabs, Inc. To generate pMXs-miR-150-GFP, miR-150 was amplified by RT-PCR from splenocyte using primers as below: miR-150 forward primer containing *Xho*I site, 5′-TCG ACT CGA GAC AGG AAC CCC CTC CCT CAG C-3′; miR-150 reverse primer with *Xho*I site, 5′-TCG ACT CGA GGG AAG GGA CCC AAG GCA TCC C-3′. The amplified PCR product was cloned into pMXs-miR-GFP/puro after treatment with *Xho*I restriction enzyme. Retroviruses were generated using TransIT transfection reagents and Platinum-E retroviral packing cell line as manufacturer’ protocol (Mirus Bio Corp., Madison, WI). To add-back miR-150 to CD8^+^ T cells, 1 × 10^6^ CD8^+^ T cells were transduced with multiplicity of infection of 10 of retro-miR-150-GFP or retro-miR-GFP with polybrene (8 μg/mL, Sigma-Aldrich) and IL-2 (10 U/mL, R&D systems). To measure the expression levels of anergy related genes after miR-150 add-back, CD8^+^ T cells were harvested at 2 d after virus infection and GFP-positive and negative populations were sorted using a FACS Aria cell sorter (BD Biosciences) and performed qPCR. For measurement of granzyme B, cyclin B1 and Blimp1, sorted GFP-positive and negative populations were incubated with anti-CD3/anti-CD28 (Life Technology) for 4 h, and PCR performed.

### TMEM20 knock-down study

For TMEM20 knockdown, isolated *mir-150* ^−/−^ naïve CD8^+^ T cells were transfected with 300 pmol of siRNA (none, control, or TMEM20: CGCTGGAGTGATACTTATCGTGAGACCAC) using Amaxa nucleofector (Lonza). At 48 hr after transfection, the cells were harvested and used to measure TMEM20 mRNA levels and intracellular Ca^2+^ levels as described above.

### Statistical analysis

Bar graphs are displayed as mean ± SEM. Statistical analyses were performed with Prism version 5.0 (GraphPad Software) using an unpaired two-tailed Student’s *t*-test or Kaplan-Meier log-rank test for survival analysis. A *p* value < 0.05 was considered statistically significant.

## Electronic supplementary material

Dataset 1-2 and Table 1-2
