# Supplementary material for: MicroRNA-150 modulates intracellular Ca2+ levels in naïve CD8+ T cells by targeting TMEM20
Source: Sci Rep. 2017 Jun 1;7:2623. doi: 10.1038/s41598-017-02697-x (PMC5453935; doi:10.1038/s41598-017-02697-x)
Supplement: Supplementary file 1 — Dataset 1-2 and Table 1-2 [file 41598_2017_2697_MOESM1_ESM.doc]

**Supplementary Information**

**MicroRNA-150 modulates intracellular Ca^2+^ levels in naïve CD8^+^ T cells by targeting TMEM20**

Tae-Don Kim^1,2,8,*^, Hong-Ryul Jung^1,3,8^, Sang-Hwan Seo^1,8^, Se-Chan Oh^1,2^, Youngho Ban^4^, Xiaoxia Tan^5^, Jung Min Kim^6^, Sang Hyun Lee^1^, Duk-Su Koh^7^, Haiyoung Jung^1^, ^2^, Young-Jun Park^1,2^, Suk Ran Yoon^1,2^, Junsang Doh^3^, Sang-Jun Ha^4^, Inpyo Choi^1,2,*^ & Philip D. Greenberg ^5,*^

***

***

**
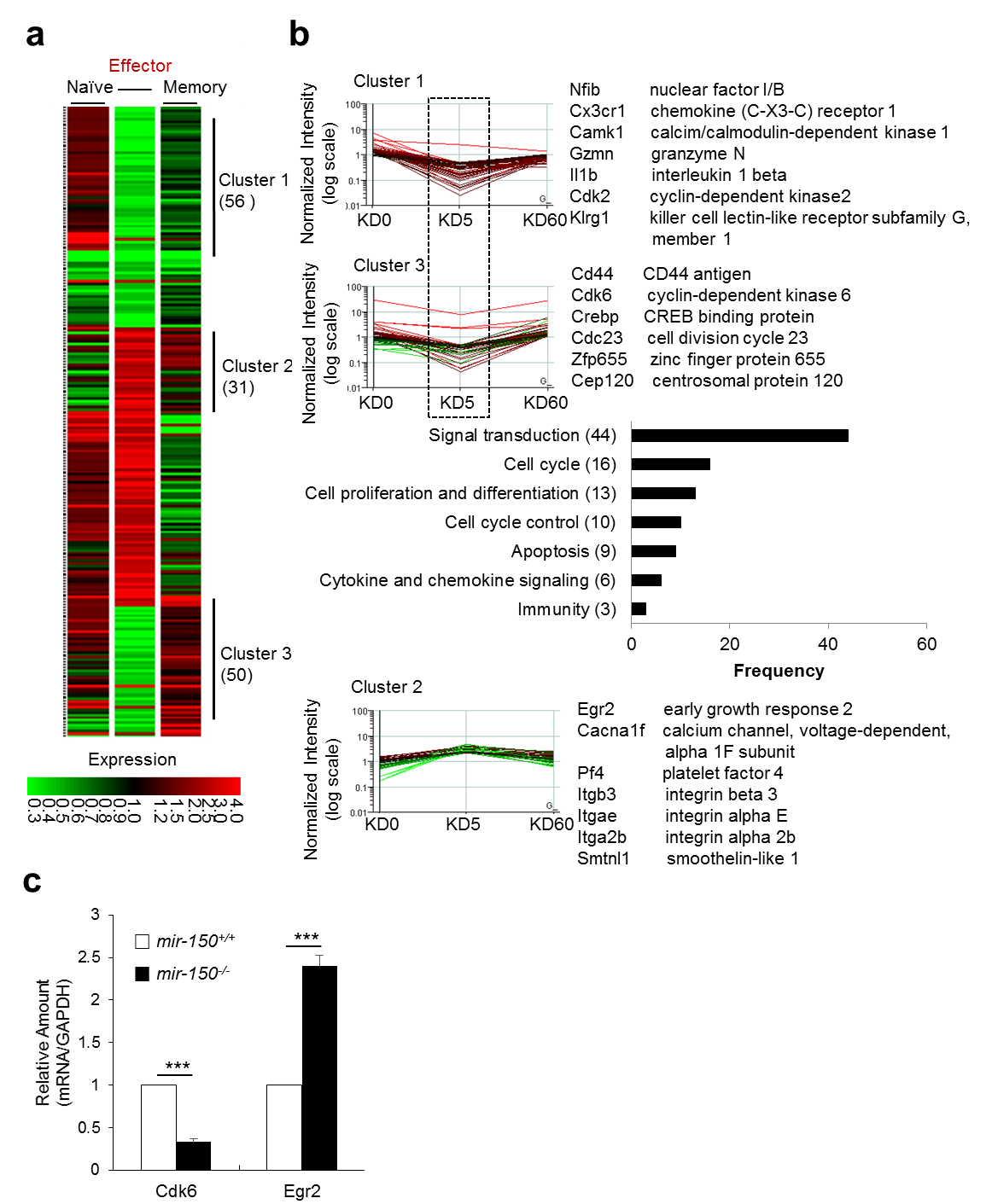
**

**Figure S1. miR-150 deficiency leads anergy-inducing gene signature in naïve CD8^+^ T cells.** (**a**) Genome-wide relative mRNA expression profiling of naïve, effector and memory CD8^+^ T cells. Colors indicate the degree of up-regulated (red) and down-regulated (green) mRNA in *mir-150^-/-^* CD8^+^ T cells compared to *mir-150^+/+^* CD8^+^ T cells. Samples were collected from spleens in the mice infected with Lm-gag as described in figure 1 legend. Naïve: before Lm-gag infection (CD8^+^CD44^lo^CD62L^hi^); effector: 5 day after Lm-gag infection (CD8^+^CD44^hi^CD62L^lo^); and memory: 50 days after Lm-gag infection (CD8^+^C44^hi^CD62L^hi^). (**b**) Classification of down-regulated genes (cluster1 and 3, top) and up-regulated genes (cluster 2, bottom) in *mir-150^-/-^* effector CD8^+^ T cells compared to *mir-150^+/+^* effector CD8^+^ T cells. (**c**) qPCR analysis for the relative expression of Cdk6 and Egr2 in *mir-150^+/+^* and *mir-150^-/-^* naïve CD8^+^ T cells. The expression level was normalized using the level of GAPDH. ***: *P* < 0.001, Data are means ± SEM of duplicate triplicate samples from a single experiment and are representative of two independent experiments.

**
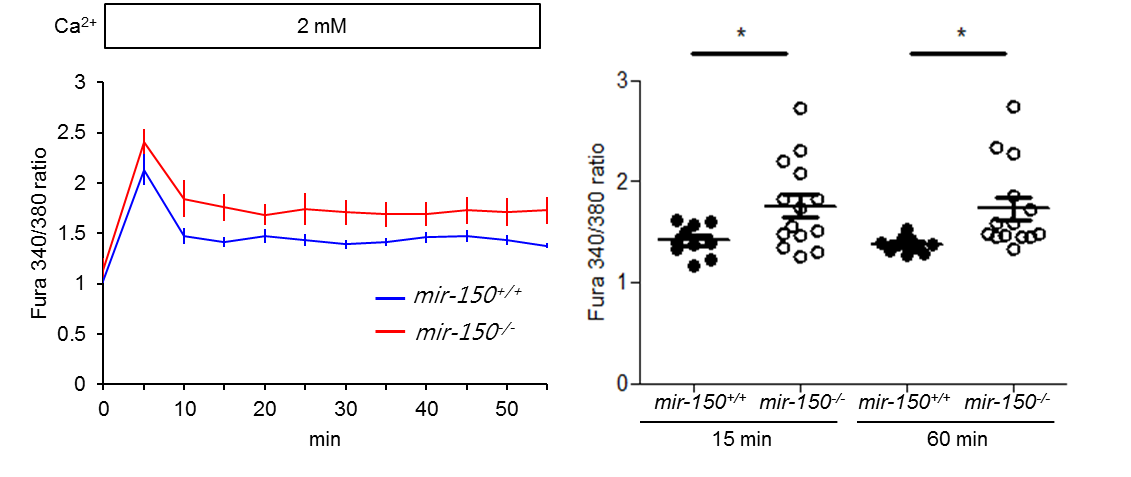
**

**Figure S2. Increased intracellular Ca^2+^ level is sustained in *mir-150^-/-^* naïve CD8^+^ T cells.**

Long-term measurement of intracellular Ca^2+^ levels in *mir-150^+/+^* or *mir-150^-/-^* naïve CD8^+^ T cells (left) and the Ca^2+^ levels at indicated time points after incubation (right).*: *P* < 0.05.

**
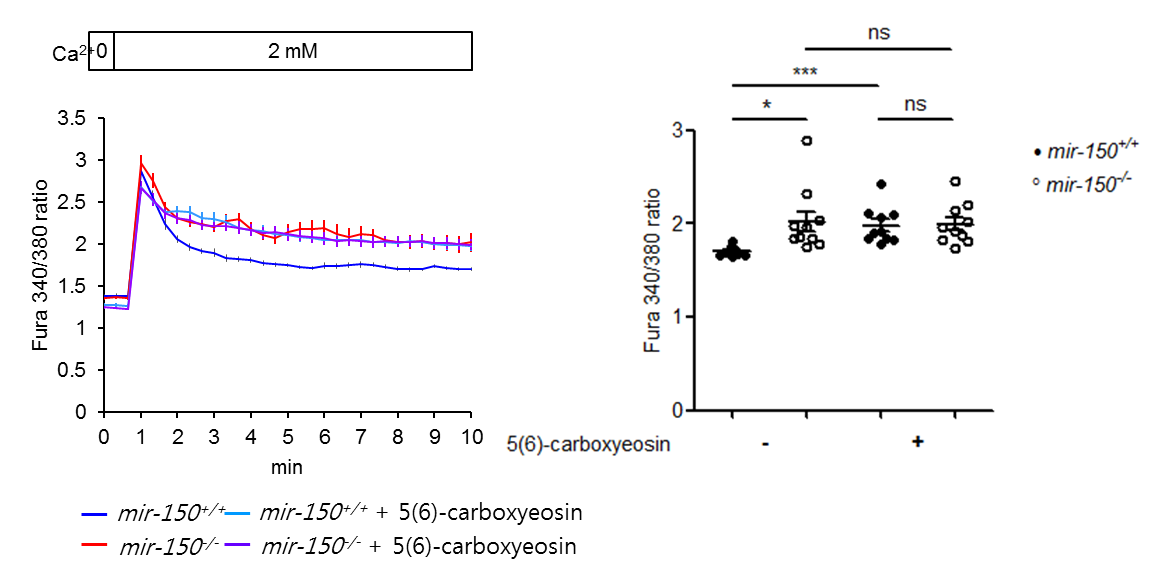
**

**Figure S3. Increased intracellular Ca^2+^ level by miR-150 deficiency is associated with PMCA in CD8^+^ T cells.** Intracellular Ca^2+^ levels in *mir-150^+/+^* or *mir-150^-/-^* naïve CD8^+^ T cells in the presence or absence of PMCA inhibitor, 5(6)-carboxyeosin (left) and the Ca^2+^ levels at 10 min after incubation (right).*: *P* < 0.05, ***: *P* < 0.001, ns: not significant.


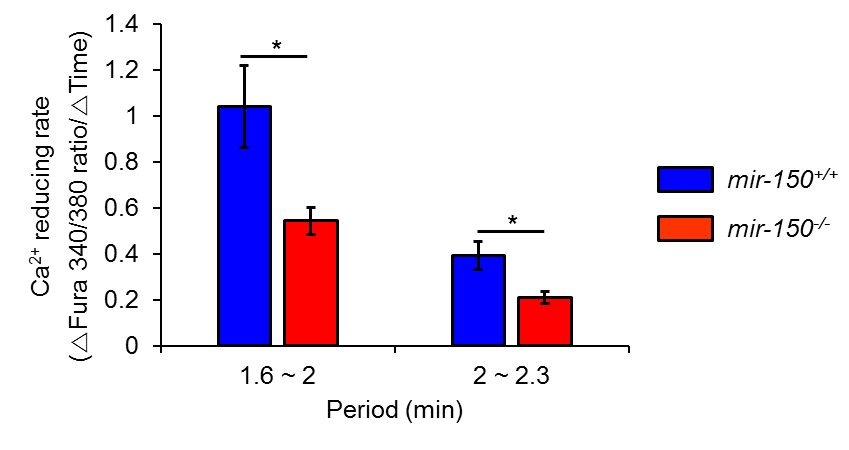


**Figure S4. Reduced Ca^2+^ reducing rates in naïve *mir-150^-/-^* CD8^+^ T cells.** Ca^2+^ reducing rates were calculated during Ca^2+^ decline periods from Fig. 4b (0 mM La^3+^ group).*: *P* < 0.05.


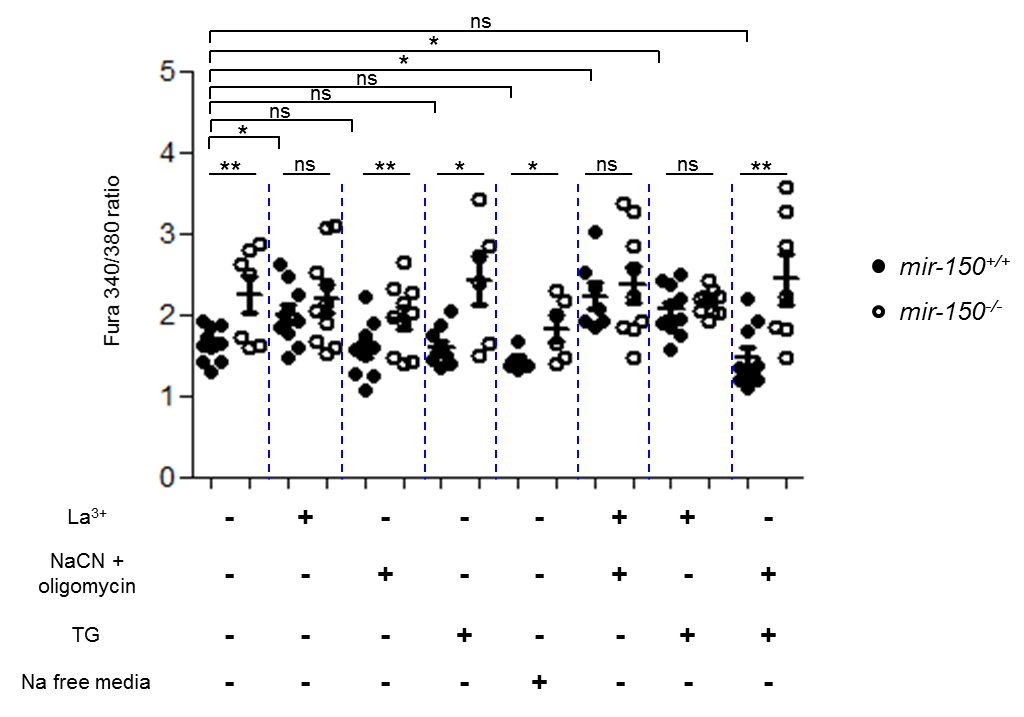


**Figure S5. Increased intracellular Ca^2+^ levels in *mir-150^-/-^* CD8^+^ T cells are not associated with SERCA, MCU, and NCX function.** Intracellular Ca^2+^ levels in *mir-150^+/+^* or *mir-150^-/-^* naïve CD8^+^ T cells in the presence of inhibitor for PMCA (1 mM La^3+^), SERCA ( 1 µM thapsigargin (TG)), MCU (3 mM sodium cyanide (NaCN) with 2 µg/mL oligomycin), or under sodium-free media and their combinations. Intracellular Ca^2+^ levels were measured at 15 min after incubation. *: *P* < 0.05, **: *P* < 0.01, ns: not significant.

**
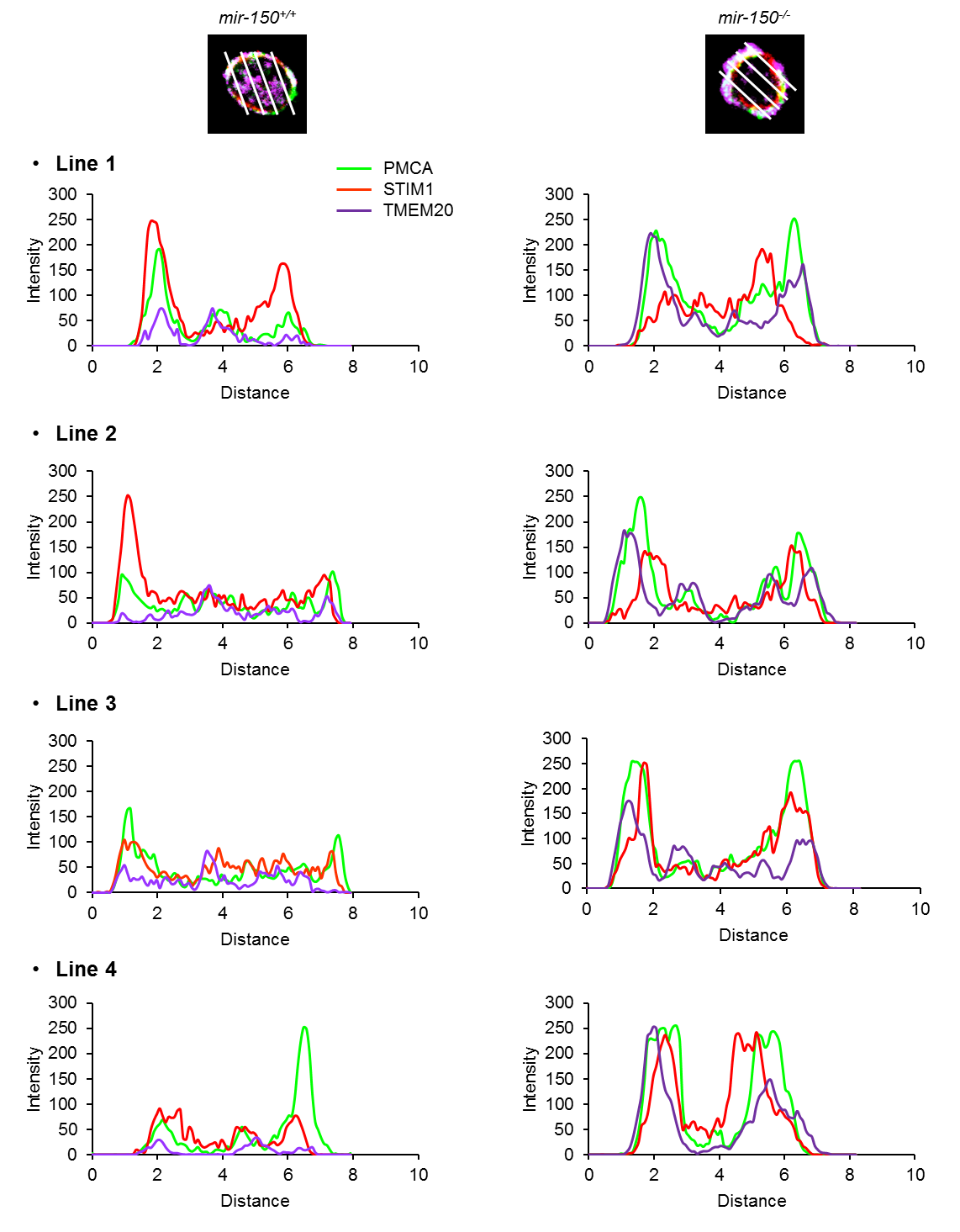
**

**Figure S6. Higher co-localization of TMEM20 with PMCA in naïve *mir-150^-/-^* CD8^+^ T cells.** Confocal microscopic analysis for co-localization of STIM1, TMEM20 and PMCA in naive *mir-150^+/+^* and *mir-150^-/-^* CD8^+^ T cells (top) and the fluorescence intensity for each molecules on the lines (down).

Table S1. Primers sequences that were used for the real-time PCR

| **Gene Name** | **Direction** | **Sequence** |
| --- | --- | --- |
| mouse TMEM20 | Forward | 5′-ttgctctctcccacactcct-3′ |
|  | Reverse | 5′-gggtcaaatgcaactgaggt-3′ |
| mouse STIM1 | Forward | 5′-GGTAGCCGAAACACACGAAT-3′ |
|  | Reverse | 5′-GAAAGGAAGGGAGGTGAAGG-3′ |
| mouse Cbl-b | Forward | 5′-TTCGGGAACCAAGCTACACCA-3′ |
|  | Reverse | 5′-CAGGCTGTAGTCCACCAGACCA-3′ |
| mouse GzmB | Forward | 5′-tcgaccctacatggccttac-3′ |
|  | Reverse | 5′-tggggaatgcattttaccat-3′ |
| NeoR | Forward | 5′-ATGACTGGGCACAACAGACA-3′ |
|  | Reverse | 5′-AGTGACAACGTCGAGCACAG-3′ |
| AANAT | Forward | 5′-CATCTGCCTCTTGGGACCT-3′ |
|  | Reverse | 5′-AGCTCTGGACACAGGGTGAG-3′ |
| mouse GAPDH | Forward | 5′-atcactgccacccagaagac-3′ |
|  | Reverse | 5′-agatccacgacggacacatt-3′ |
| mouse Egr2 | Forward | 5′-CCTCCACTCACGCCACTCTC-3′ |
|  | Reverse | 5′-CACCACCTCCACTTGCTC-3′ |
| mouse p27 | Forward | 5′-ccgaggaggaagatgtcaaa-3′ |
|  | Reverse | 5′-aaattccacttgcgctgact-3′ |
| mouse Bcl2 | Forward | 5′-CTGCACCTGACGCCCTTCACC-3′ |
|  | Reverse | 5′-CACATGACCCCACCGAACTCAAAGA-3′ |
| mouse cyclinB1 | Forward | 5′-atctccgacaactggaggaa-3′ |
|  | Reverse | 5′-tcttcttgggcacacaactg-3′ |
| mouse cMyc | Forward | 5′-tctccactcaccagcacaac-3′ |
|  | Reverse | 5′-gttcctcctctgacgttcca-3′ |
| mouse IL-2 | Forward | 5′-cccacttcaagctccacttc-3′ |
|  | Reverse | 5′-ttcaattctgtggcctgctt-3′ |
| mouse DUSP1 | Forward | 5′-aggacaaccacaaggcagac-3′ |
|  | Reverse | 5′-gaggtaagcaaggcagatgg-3′ |
| mouse DUSP6 | Forward | 5′-tgtccccattccttcagttc-3′ |
|  | Reverse | 5′-agcaaatctctccctccgtaa-3′ |
| mouse IFNγ | Forward | 5′-aactggcaaaaggatggtga-3′ |
|  | Reverse | 5′-gacctgtgggttgttgacct-3′ |
| mouse SOCS2 | Forward | 5′-GCCATCAATGACCCCTTCATT-3′ |
|  | Reverse | 5′-GCTCCTGGAAGATGGTGGTGATGG-3′ |
| mouse EOMES | Forward | 5’-GCCTACCAAAACACGGATA-3’ |
|  | Reverse | 5’-TCTGTTGGGGTGAGAGGAG-3’ |
| mouse T-bet | Forward | 5’-GTTCCCATTCCTGTCCTTC-3’ |
|  | Reverse | 5’-CCTTGTTGTTGGTGAGCTT-3’ |
| mouse Blimp | Forward | 5’-ACACCGGGACTCCTACTCCT-3’ |
|  | Reverse | 5’-ACTCGGTAGGGAAGCTGGAT-3’ |
| mouse DGKα | Forward | 5’-CAACATGCAAAAAGCTGGAA-3’ |
|  | Reverse | 5’-GTGATTATTTTGGCCGCACT-3’ |
| mouse DGKζ | Forward | 5’-AGGAGAATGGGGAGACCTGT-3’ |
|  | Reverse | 5’-TCACGCTGGATCATCTGGTA-3’ |
| mouse SHP-1 | Forward | 5’-GGTGGTACGGTTTGGAGAGA-3’ |
|  | Reverse | 5’-ATGCTGAGCCTCTGTGGTCT-3’ |

Table S2. The following oligonucleotides were used to generate AANAT reporter plasmids containing the 3' UTR of mouse TMEM20

| **Inserts** | **Direction** | **Sequence** |
| --- | --- | --- |
| mouse TMEM20 3′ UTR | Forward | 5′-AAGAATTCTGAAGTGTCACTGCTGA-3′ |
|  | Reverse | 5′-AACTCGAGAGTTGTAAAACCCAACA-3′ |

Flanking sequences for cloning purposes are underlined.
